# A Full Range Experimental Study of Amplitude- and Frequency-Dependent Characteristics of Rubber Springs

**DOI:** 10.3390/polym14214662

**Published:** 2022-11-01

**Authors:** Yanping Shi, Juanjuan Li, Yuan Wang, Xuebing Li, Yuanjing Gao, Dong Zhao, Baohui Shi, Lihua Zou, Xiuduo Song, Yuanyuan Shang

**Affiliations:** 1 College of Textiles and Clothing, Qingdao University, Qingdao 266071, China; 2 Key Laboratory of Clean Dyeing and Finishing Technology of Zhejiang Province, Shaoxing 310063, China; 3 Collaborative Innovation Center for Eco-Textiles of Shandong Province and the Ministry of Education, Qingdao University, Qingdao 266071, China; 4 Department of Automotive Engineering, Tsinghua University, Beijing 100084, China; 5 FEIBA Technology (Shenzhen) Co., Ltd., Shenzhen 518000, China; 6 Key Laboratory of High Performance Fibers & Products, Ministry of Education, Donghua University, Shanghai 201620, China; 7 School of Textile and Garment, Anhui Polytechnic University, Wuhu 241000, China; 8 Xi’an Modern Chemistry Reserch Institute, Xi’an 710065, China

**Keywords:** vehicle suspension, rubber spring, dynamic stiffness, loss factor, Payne effect

## Abstract

This paper provides a comprehensive understanding of the amplitude- and frequency-dependent characteristics of rubber springs. The dynamic nonlinear inelasticity of rubber is a key academic problem for continuum mechanics and a bottleneck problem for the practical use of rubber structures. Despite intensive efforts witnessed in industrial applications, it still demands an unambiguous constitutive model for dynamic nonlinear inelasticity, which is known as the Payne effect. To this end, three types of rubber springs (shear-type (ST), compression-type (CT) and shear-compression-combination-type (SCCT)) were tested with amplitude and frequency sweeps in different conditions. We investigated and present changes in dynamic stiffness and loss factor with amplitude, frequency and the hysteresis loops of different rubber springs. We also propose a hypothesis and research strategy to study a constitutive model involving multiple factors of hyperelasticity, the Mullins effect, viscoelasticity and the Payne effect, which we hope will provide new ideas for the establishment of a constitutive equation.

## 1. Introduction

Rubber springs (also known as rubber-to-metal parts) are widely used in vehicles for suspension or flexible connections between two components because of their excellent anti-vibration and noise reduction performance [1] compared to parts composed of traditional polymers [2]. The dynamic property of rubber springs is one of the most concerning aspects for vehicle system designers [3], as an optimized design cannot be achieved without a comprehensive understanding of the dynamic characteristics of rubber springs [4,5]. However, because of the special microstructure of carbon-filled rubber, the dynamic characteristic of rubber springs is complicated, which strongly correlates to the Payne effect [6,7,8] (sometimes known as the Fletcher–Gent effect), and is not well understood [9].

**Figure 1:**
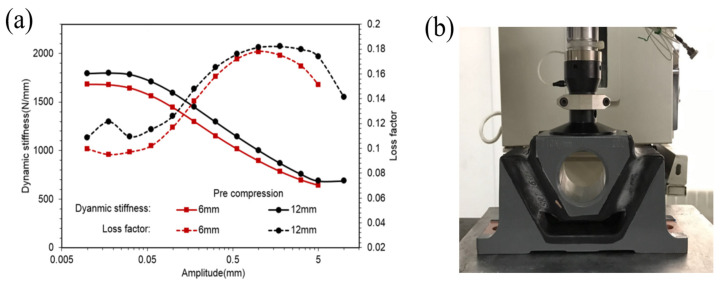
Dependency of dynamic stiffness and loss factor on the amplitude (**a**) of a rubber spring (**b**).

Payne intensively studied the Payne effect [6,7,8], which manifests that an increase in strain amplitude will lead to a decrease in storage modulus and a maximum in loss factor, as in trends shown in Figure 1. Physically, the Payne effect can be attributed to the deformation-induced changes in materials’ microstructures, i.e., the breaking or reforming of weak physical bonds within filler aggregates [10]. Previous models were validated in perfect agreement with the Payne effect. However, it is difficult to extend them for industrial applications as they will mostly lose effect on the occasion of three-dimensional (3D) continuum mechanics, especially for finite element simulations [11,12]. The solutions to recreating amplitude dependence possibly include incorporating internal variables with an intrinsic time scale [13,14], as well as non-linear viscoelastic evolution laws relating to both the current strain and stress [15,16]. Nevertheless, it has proven difficult to capture the storage modulus tandem with loss factor over a wide range of strain amplitudes and frequencies with the same parameters [17]. The concept of plasticity was also used for modeling the Payne effect [18,19]; the predicted results showed poor agreement with large strain situations. Another alternative approach to modeling the Payne effect is introducinge a single smooth frictional element in parallel with an elastic spring [20,21,22]. Differing from Coulomb friction, a smooth frictional element evolves smoothly from a stationary state to stable slipping. Perfect agreement was found using this method for cases of small strains, but prediction was ineffective for large strains [23,24]. Recently, Donner et al. [25] moved one step closer to a solution by involving rate-dependent behavior with a time-rescaling invariance method; however, a completely applicable regime still demands more research.

**Figure 2:**
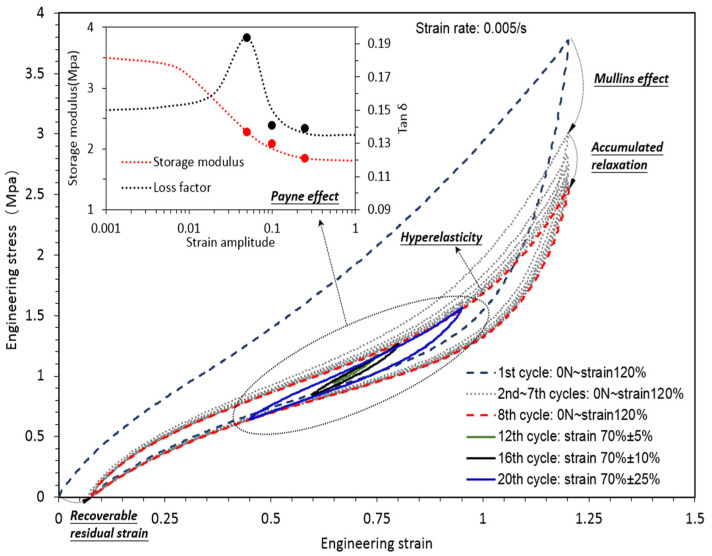
Inelasticity of CBFR.

To explain the inelastic behavior of CBFR, we carried out a representative strain–stress curve obtained from a quasi-static cyclic uniaxial tension test, which is displayed in Figure 2. Noticeable stress softening (also known as the Mullins effect) was found between the first and second cycle of 0N~120% strain. The stress at 120% strain gradually decreased to an asymptotic value, which can be regarded as accumulated relaxation, also known as viscoelasticity. Different oscillation levels in the nominal strain of 70% resulted in different storage modulus and loss factors, which corresponded to the Payne effect. Intensive efforts have been dedicated to hyperelasticity, the Mullins effect and viscoelasticity, resulting in many rubber engineering constitutive models depicting their interconnections. Nevertheless, the critical Payne effect highlighting the strong inelasticity of CBFR has not been well understood, and a systematic constitutive model is still not available for engineering application.

In a vehicle system, rubber springs sometimes work with a high frequency and a small amplitude; at other times they work with a low frequency and a large amplitude, e.g., the primary suspensions of high-speed trains, in which the dynamic behavior of the vehicle system shows considerable differences [3,26] because of the amplitude- and frequency-dependent characteristics of rubber springs. Therefore, an amplitude- and frequency-dependent model is necessary for vehicle dynamic simulation; a comprehensive experimental study of different types of rubber springs is the foundation for understanding their dynamic characteristics. To understand the amplitude- and frequency-dependent characteristics of rubber springs, it is necessary to carry out special tests, e.g., a wide range of amplitude sweep tests in cases of quasi-static (very low frequency) conditions, a wide range of frequency sweep tests under small amplitudes, and combinations of the two. In addition, rubber springs sometimes work with large deflections; therefore, geometric nonlinearity should be considered. Berg [20,21,22] presented testing results of two rubber springs without considering a preload. There were only three or four measured points for amplitude and frequency sweep, which was not sufficient to describe amplitude- and frequency-dependent characteristics (the types of rubber springs used were not mentioned). Sjöberg [27] presented test results for cylindrical rubber springs composed of two rubber mixtures under a static preload of 3.5 kN. The minimum frequency and amplitude of these tests were 1 Hz and 0.05 mm, respectively. The height of the cylindrical rubber springs was 70 mm, and the maximum amplitude of the test was 3 mm for the hard specimen and 4 mm for the soft specimen, respectively, which means that geometric nonlinearity was ignored. In another Sjöberg study [28], a rubber spring with a height of 20 mm was tested using a maximum amplitude of approximately 1.2 mm; geometric nonlinearity was not considered. This is why all the hysteresis loops from tests in his study were symmetric. Gil-Negrete [29] presented test results for a simple shear specimen without a transversal preload. The amplitude sweep test was performed using a 102 Hz frequency, which was not a quasi-static test. Kaldas et al. [30] presented test results for three rubber mounts using a 0.1 Hz frequency and only five amplitudes. Shi and Wu [31] presented test results for a rubber mount used on a high-speed train. The maximum frequency was 8 Hz for the frequency sweep and the minimum frequency was 1 Hz for the amplitude sweep. Sedlaczek et al. [32] presented test results for a rubber bushing using a frequency sweep, but the amplitude sweep was not studied. Alonso et al. [33] presented test results for a rubber bushing, in which the minimum frequency for the amplitude sweep was 1 Hz. Dzierek [34] presented test results for a rubber bushing. The minimum frequency for the amplitude sweep was 10 Hz. All the above-mentioned studies delivered a level of awareness regarding the complicated dynamic characteristics of rubber springs; however, it is necessary to carry out a full range of amplitude and frequency sweeps for different types of rubber springs [4,35,36]. 

In this study, three types of rubber springs (shear-type (ST), compression-type (CT) and shear-compression-combination-type (SCCT)) were tested using a full range of amplitude and frequency sweeps. Dynamic stiffness and loss factor against amplitude and frequency, as well as the shapes of hysteresis loops for different types of rubber springs, were examined. A hypothesis and research strategy or experimental idea is proposed to study a constitutive model involving multiple factors of hyperelasticity, the Mullins effect, viscoelasticity and the Payne effect, which we hope will provide new ideas and research directions towards the establishment of a constitutive equation. 

## 2. Rubber Spring Samples

Rubber springs were prepared using natural rubber (SMR20) purchased from China Petrochemical Corporation Beijing Yanshan Company. The neodymium used was cis-1,4-polybutadiene rubber (BUNA CB24), from German Lang-sheng Chemical Co., Ltd,Qingdao, China. Carbon black (N550) was obtained from Cabot Co., Ltd, Shanghai, China. Other additives were commercially available industrial products. The detailed experiment formula is shown in Table 1.

**Figure 3:**
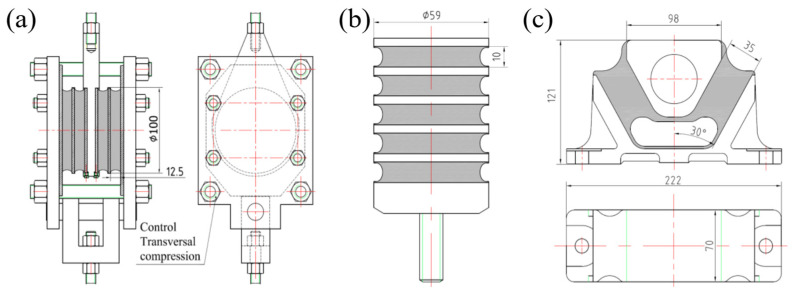
Layouts of rubber spring samples: (**a**) ST, (**b**) CT and (**c**) SCCT.

Three types of rubber springs were tested. Figure 3 shows the layout of ST, CT and SCCT rubber springs. The ST rubber spring test was conducted by clamping two ST rubber springs using two rigid metal plates with four bolts, which were also used to control transversal compression. Each ST rubber spring had two rubber layers, was 12.5 mm high and had a Φ 100 mm diameter. Each CT rubber spring had five rubber layers, was 10 mm high and had a 59 mm diameter. Each SCCT rubber spring had two pieces of 35 mm thick rubber and a 30-degree angle against the vertical line; other dimensions are illustrated in Figure 3c.

## 3. Experiments

Amplitude and frequency sweep tests were carried out on all three types of rubber springs introduced in Section 2. The testing conditions were as follows:

- ST rubber spring:
  1. Amplitude sweeps from 0 to the maximum of 17.8 mm with 0.05, 1 and 20 Hz frequencies under 0 and 4 mm transversal compression.
  2. Frequency sweeps from 0 to the maximum of 31.6 Hz with 0.01, 0.1 and 1 mm amplitudes under 0 and 4 mm transversal compression.
- CT rubber spring:
  1. Amplitude sweeps from 0 to the maximum of 3.15 mm with 0.05, 1 and 10 Hz frequencies under 1, 4 and 6 mm pre-compression.
  2. Frequency sweeps from 0 to the maximum of 31.6 Hz with a 0.01 mm amplitude under 1, 4 and 6 mm pre-compression.
- SCCT rubber spring:
  1. Amplitude sweeps from 0 to the maximum of 10 mm with 0.05, 1 and 5 Hz frequencies under 6 and 12 mm pre-compression.
  2. Frequency sweeps from 0 to the maximum of 31.6 Hz with 0.01 and 1 mm amplitudes under 12 mm pre-compression.

All tests were carried out using the MTS 831.50 dynamic testing system; the excitation applied to the samples were sinusoidal waveforms. The environment temperature of the laboratory was 23 ± 2 degrees Celsius; tested samples were kept in the laboratory 24 h before testing. During the testing process, we monitored the temperature of the samples using an infrared thermometer. If the temperature increased by more than 1 degree Celsius, the test was suspended until the temperature returned to the specified temperature range. 

## 4. Experimental Results

It is well known that the rubber spring is not perfectly elastic, which means that a force response to an applied sinusoidal displacement on a rubber spring has the same frequency but is shifted to a phase called a loss angle. The dynamic response of a rubber spring is usually expressed as a complex stiffness $K^{*}=K_{Stor}+iK_{Loss}=K_{Stor}\left(1+i\tan\delta \right)$, where $K_{Stor}$ and $K_{Loss}$ represent the in-phase storage and out-of-phase loss stiffness, respectively, and $i=\sqrt{-1}$. The magnitude of complex stiffness $\left|K^{*}\right|=\sqrt{K_{Stor}^{2}+K_{Loss}^{2}}$ is defined as dynamic stiffness, and the ratio $K_{Loss}/K_{Stor}$ is denoted as a loss factor. Dynamic stiffness and loss factor changes with amplitude and frequency are presented and analyzed in this section.

**Figure 4:**
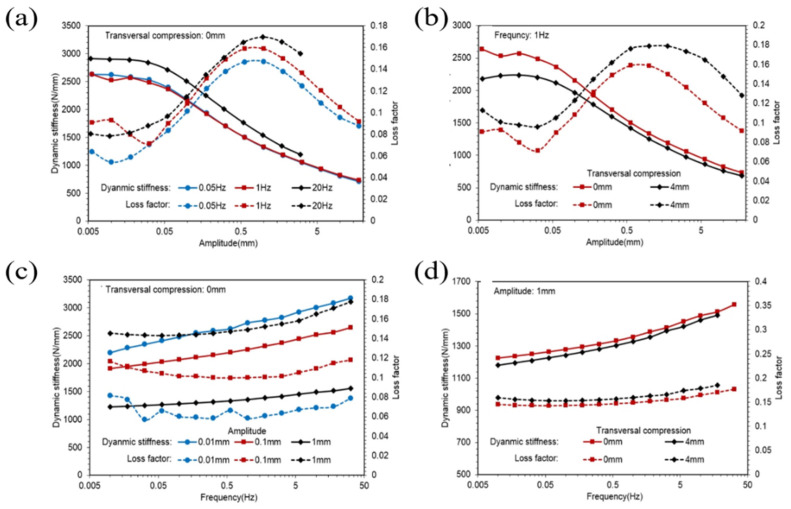
Dynamic stiffness and loss factors curves of the ST rubber spring. (**a**) Dynamic stiffness and loss factors of the ST rubber spring with amplitudes of different frequencies. (**b**) Dynamic stiffness and loss factors of the ST rubber spring against the am-plitude in different transversal compressions. (**c**) Dynamic stiffness and loss factors of the ST rubber spring against the frequency in different amplitudes. (**d**) Dynamic stiffness and loss factors of the ST rubber spring with transversal compression in different amplitudes.

Figure 4a shows changes in dynamic stiffness and loss factors of the ST rubber spring with amplitudes of different frequencies. Figure 4b illustrates changes in dynamic stiffness and loss factors of the ST rubber spring against the amplitude in different transversal compressions. Figure 4c plots dynamic stiffness and loss factors of the ST rubber spring against the frequency in different amplitudes. Figure 4d shows dynamic stiffness and loss factors of the ST rubber spring with transversal compression in different amplitudes.

**Figure 5:**
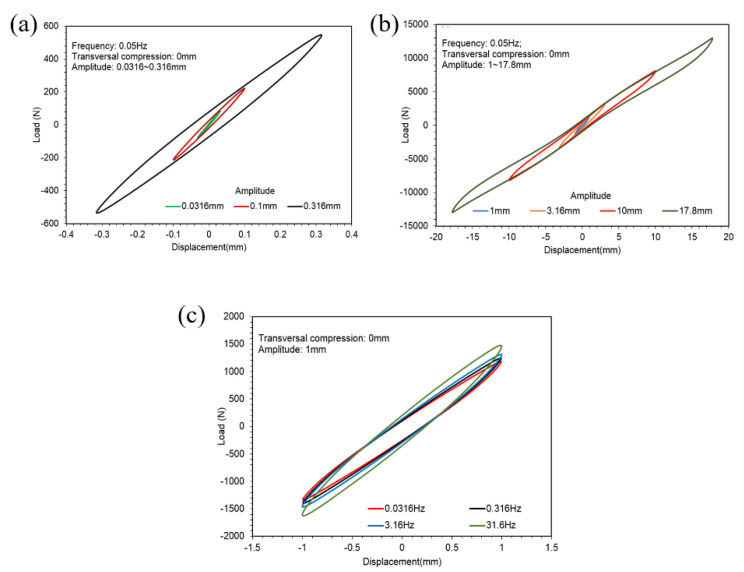
Hysteresis loops of the ST rubber spring. (**a**,**b**) Hysteresis loops of different amplitudes of the ST rubber spring in 0.05 Hz without transversal compression. (**c**) Hysteresis loops under different frequencies in amplitude 1 mm without transversal compression.

Figure 5a,b plot hysteresis loops of different amplitudes of the ST rubber spring in 0.05 Hz without transversal compression. Figure 5c shows hysteresis loops under different frequencies in amplitude 1 mm without transversal compression.

**Figure 6:**
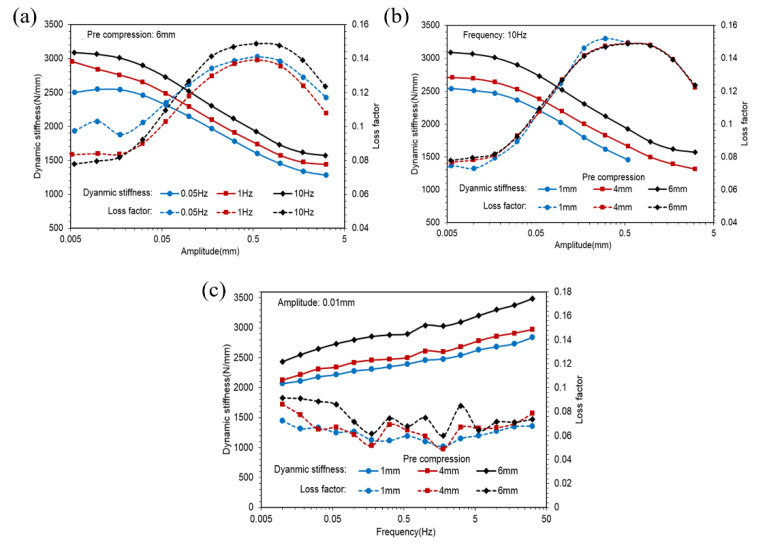
Dynamic stiffness and loss factors curves of the CT rubber spring. (**a**) Dynamic stiffness and loss factors of the CT rubber spring against the amplitude in different frequencies. (**b**) Dynamic stiffness and loss factors of the CT rubber spring against the amplitude in different pre-compression. (**c**) Dynamic stiffness and loss factors of the CT rubber spring against the frequency in different amplitudes.

Figure 6a shows dynamic stiffness and loss factors of the CT rubber spring against the amplitude in different frequencies. Figure 6b illustrates dynamic stiffness and loss factors of the CT rubber spring against the amplitude in different pre-compression. Figure 6c shows dynamic stiffness and loss factors of the CT rubber spring against the frequency in different amplitudes. 

**Figure 7:**
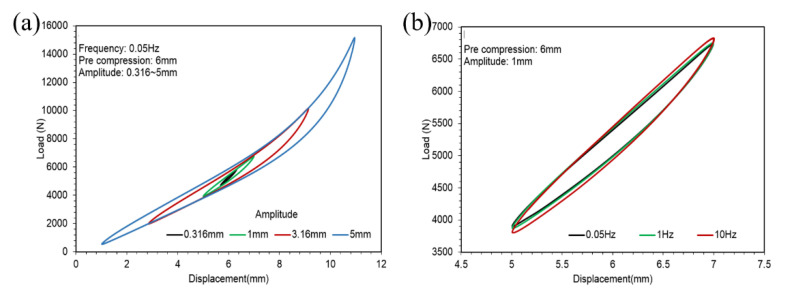
Hysteresis loops of the CT rubber spring. (**a**) Hysteresis loops of different amplitudes of the ST rubber spring in 0.05 Hz with 6 mm pre-compression. (**b**) Hysteresis loops of different frequencies in a 1 mm amplitude with 6 mm pre-compression.

Figure 7a shows the hysteresis loops of different amplitudes of the ST rubber spring in 0.05 Hz with 6 mm pre-compression. Figure 7b plots the hysteresis loops of different frequencies in a 1 mm amplitude with 6 mm pre-compression.

**Figure 8:**
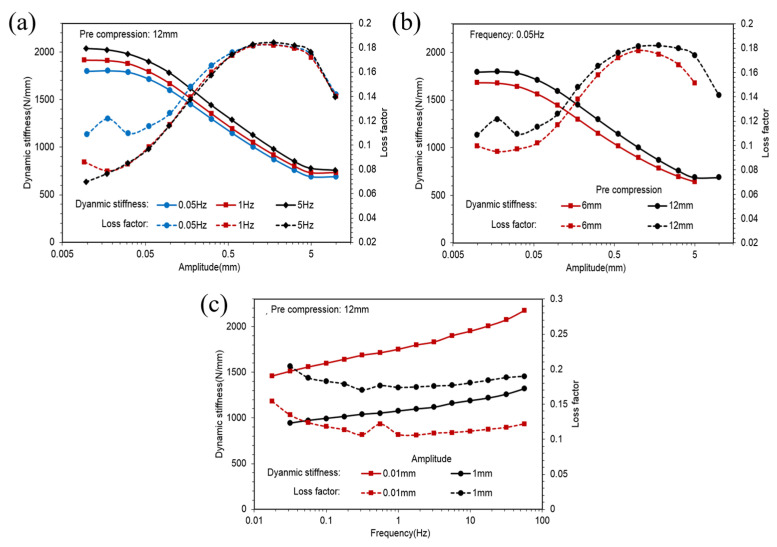
Dynamic stiffness and loss factors curves of the SCCT rubber spring.

Figure 8a shows dynamic stiffness and loss factors of the SCCT rubber spring against the amplitude in different frequencies. Figure 8b shows dynamic stiffness and loss factors of the ST rubber spring against the amplitude in different pre-compressions. Figure 8c plots dynamic stiffness and loss factors of the SCCT rubber spring with pre-compression in different amplitudes.

**Figure 9:**
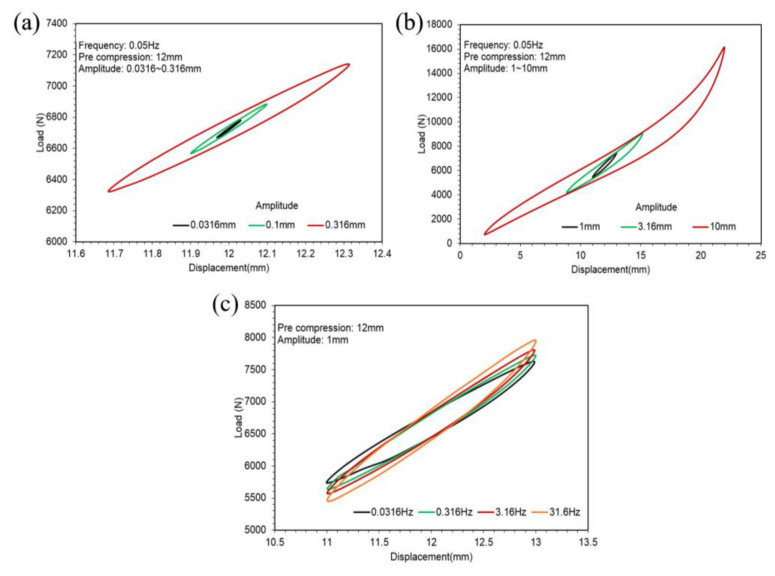
Hysteresis loops of the SCCT rubber spring.

Figure 9a,b show the hysteresis loops of different amplitudes of the SCCT rubber spring in 0.05 Hz with 12 mm pre-compression. Figure 9c illustrates the hysteresis loops of different frequencies in 1 mm amplitude with 12 mm pre-compression.

Observing Figure 3a,b, it is evident that dynamic stiffness was almost constant in very small amplitudes and decreased to an asymptotic value with an increase in amplitude, whereas loss factors were almost constant in very small amplitudes, increased to a peak value first, then decreased to an asymptotic value with an increase in amplitude. Figure 6a,b and Figure 8a,b illustrate similarities between CT and SCCT rubber springs. This phenomenon is known as the Payne effect. Observing Figure 3c,d, we find that dynamic stiffness increased linearly with the frequency in a logarithmic coordinate system; this means that the relationship between dynamic stiffness and frequency was a logarithmic scale. Additional similarities were found between CT and SCCT rubber springs, as indicated in Figure 6c and Figure 8c. Observing Figure 3b,d, we find that transversal compression led to decreased dynamic stiffness but increased loss factors. Observing Figure 6c and Figure 8c, we find that pre-compression resulted in increases in both dynamic stiffness and loss factors. 

Comparing the hysteresis loops shown in Figure 5, Figure 7 and Figure 9, we find that the hysteresis loops of the ST rubber spring were symmetric, whereas the hysteresis loops of CT and SCCT rubber springs were asymmetric. This is because of the structural difference between the rubber springs. Hysteresis loops for all three rubber spring types show nonlinearity in large amplitude; however, the nonlinearity of the ST rubber spring was lower than that of CT and SCCT rubber springs, because the geometric nonlinearity of CT and SCCT springs was stronger than that of the ST rubber spring in a large amplitude. 

## 5. Research Ideas Regarding Model Establishment

### 5.1. Research Questions/Hypotheses

Here, we share our research ideas and experimental plans for the construction of the constitutive model, hoping to provide new research ideas for relevant research.

**Figure 10:**
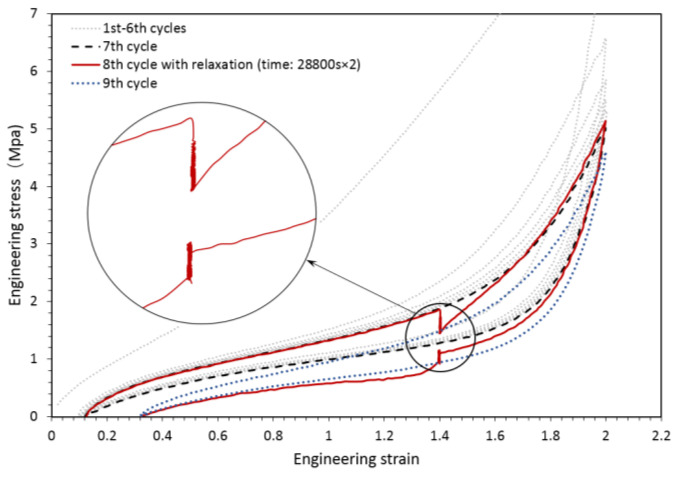
Cyclic tests with relaxation on CBFR.

**Figure 11:**
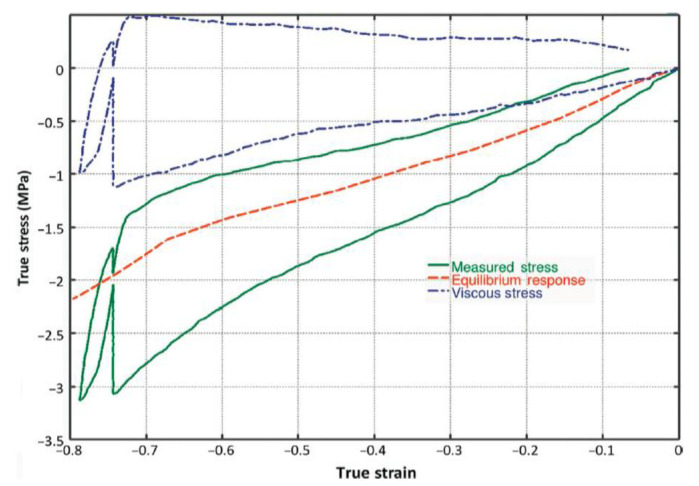
The strain was held constant for 10 min at a true strain of −0.75. The experimentally measured stress (green solid line) was decomposed into equilibrium stress (red dashed line) and viscoelastic stress (blue dash-dot line) (extracted from Bergström’s book [38]).

First, we assumed that the essence of addressing the Payne effect in a constitutive model was to simulate the static hysteresis as shown in Figure 10, which was caused by “smooth internal friction” of materials, including filler–filler, molecule–filler and molecule–molecule friction. This means that most of the dissipated energy in a quasi-static hysteresis loop was caused by “internal friction” rather than viscosity. A cyclic quasi-static uniaxial tension with a long-time relaxation test was conducted on a CBFR to verify our assumption. Differing from a similar test carried out by Bergström [37] (see Figure 11), which showed that relaxation stress at a certain strain in both loading and unloading stages tends to an equilibrium response, our test indicated that relaxation stress in the unloading stage first approaches, but subsequently departs from, the stable relaxation stress in the loading stage. We suggest that this can be attributed to a shorter relaxation time (~10 min) in Bergström’s test [37]. Our results revealed that static hysteresis loops were not solely caused by viscoelasticity. There must be another potential mechanism dominating quasi-static hysteresis loops, which we called “smooth internal friction”.

However, we need to expand our postulations regarding the following:

(a) Internal friction evolves differently with respect to the deformation level for large strains, which was the major difference compared with that of small strains.
(b) A governing equation describing the dependence of internal friction on specific evolution variables needs to be developed to determine which variables (e.g., strain energy density, maximum largest principal stretch, invariants or maximal principal stress, etc.) will dominate the evolution of internal friction in different deformation levels.
(c) The constitutive framework of CBFR’s complicated inelasticity can be established by incorporating comprehensive factors of hyperelasticity, the Mullins effect, viscoelasticity and internal friction. Numerical implementation can be realized using the finite element method based on the user subroutine of commercial finite element software.

### 5.2. Research Design

**Figure 12:**
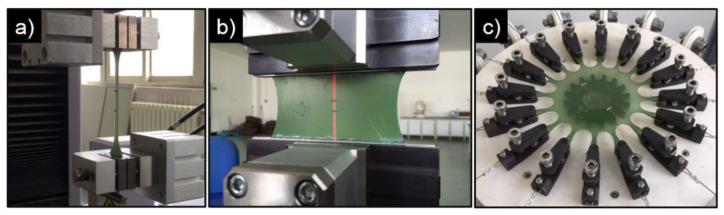
Homogeneous finite strain experimental equipment: (**a**) uniaxial tension, (**b**) planar tension (pure shear) and (**c**) equibiaxial tension.

To clearly verify the hypothesis proposed above, we plan to conduct the following investigations:

Step 1. Various homogeneous finite strain experiments, including uniaxial tension, planar tension (pure shear) and equibiaxial tension, will be carried out on CBFR. Figure 12 shows photos of different experimental equipment. All strain measurements will be conducted via laser extensometer. The purpose of these experiments is, on the one hand, to investigate the evolution of internal friction varying with different strain levels on different deformation modes and, on the other hand, to provide a detailed dataset for constitutive modeling.

Step 2. Explore which mechanical quantity (e.g., strain energy density, maximum largest principal stretch, invariants or maximal principal stress, etc.) yields the best fit between the internal friction governing equation and various experimental datasets obtained from step 1. 

Step 3. The proposed internal friction governing equation will be developed into a three-dimensional constitutive law based on the finite deformation theory of continuum mechanics. Next, the second-order Cauchy stress tensor and the fourth-order tangent elasticity tensor of internal friction will be deduced.

**Figure 13:**
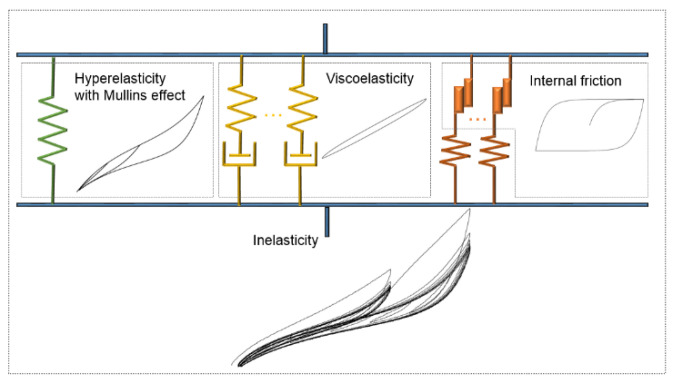
Rheological model of the inelasticity of CBFR.

Step 4. The total inelastic Cauchy stress tensor and tangent elasticity tensor will be obtained by superposing hyperelasticity with the Mullins effect, viscoelasticity and internal friction, as shown in Figure 13. Notably, the rheological model in Figure 13 is just a preliminary proposal; the final model will be elaborated after intensive experimental and theoretical study in this research.

Step 5. The proposed constitutive model will be implemented in finite element software via user subroutine.

Step 6. Validation will be accomplished by simulating the inelastic behavior of a CBFR component compared with experimental results.

## 6. Summary

Three different types of rubber springs were tested in a full range of amplitude and frequency sweeps. Changes in dynamic stiffness and loss factor with different amplitudes and frequencies, and the hysteresis loops of different rubber springs, were analyzed. The principal conclusions are:

For both transversal compression of the ST rubber spring and pre-compression of CT and SCCT rubber springs, dynamic stiffness was almost constant in very small amplitudes, and decreased to an asymptotic value with an increase in amplitude. In contrast, loss factors were almost constant in very small amplitudes, increased to a peak value first, then decreased to an asymptotic value with an increase in amplitude, which is known as the Payne effect. The relationship between dynamic stiffness and frequency was in a logarithmic scale for all types of rubber springs. The transversal compression of ST rubber springs resulted in dynamic stiffness decreasing. The pre-compression of CT and SCCT rubber springs resulted in an increase in dynamic stiffness. The structural differences between rubber springs resulted in the hysteresis loops of the ST rubber spring being symmetric, whereas the hysteresis loops of CT and SCCT rubber springs were asymmetric. Hysteresis loops for all three types of rubber springs showed nonlinearity in large amplitudes, but the nonlinearity of the ST rubber spring was lower than that of CT and SCCT rubber springs because the geometric nonlinearity of CT and SCCT rubber springs was stronger than the ST rubber spring in a large amplitude. Relaxation stress in the unloading stage first approached, but subsequently departed from, the stable relaxation stress in the loading stage. The static hysteresis loop was not solely caused by viscoelasticity; there must be another potential mechanism dominating the quasi-static hysteresis loop. A research design regarding a constitutive model involving multiple factors of hyperelasticity, the Mullins effect, viscoelasticity and the Payne effect was proposed, and we hope it provides new ideas or research directions for relevant future studies.

## Figures and Tables

**Table 1:** Experiment formula.

Item	
SMR20	70
CB24	30
N550	30
Si69	3
ZnO	5
MgO	0.3
SA	3
4020	1.5
RD	1.5
Vulcanizing agent	2
Sulfur promoter	1

## Data Availability

Not applicable.
